# Brain-Derived Neurotrophic Factor-Mediated Cognitive Impairment in Hypothyroidism

**DOI:** 10.7759/cureus.23722

**Published:** 2022-04-01

**Authors:** U Madhusudhan, Kalpana M, Vidya Singaravelu, Vidya Ganji, Nitin John, Archana Gaur

**Affiliations:** 1 Physiology, All India Institute of Medical Sciences, Bibinagar, Hyderabad, IND; 2 Pediatrics, Malla Reddy Institute of Medical Sciences, Hyderabad, IND

**Keywords:** p75ntr, neurotrophin, thyroid disorder, memory, cognition

## Abstract

Brain-derived neurotrophic factor (BDNF), which is expressed at high levels in the limbic system, has been shown to regulate learning, memory and cognition. Thyroid hormone is crucial for brain development. Hypothyroidism is a clinical condition in which thyroid hormones are reduced and it affects the growth and development of the brain in neonates and progresses to cognitive impairment in adults. The exact mechanism of how reduced thyroid hormones impairs cognition and memory is not well understood. This review explores the possible role of BDNF-mediated cognitive impairment in hypothyroid patients.

## Introduction and background

Thyroid disorders have been among the most commonly diagnosed disorders in the African population. In developed countries, the prevalence of subclinical hypothyroidism is about 8% in women and 3% in males [1]. Iodine deficiency disorders (IDD) are the commonest cause of thyroid disorder in the continent not only due to iodine status but also due to selenium deficiency thiocyanate toxicity [1,2]. The prevalence rate of thyroid disorders was found to be 1.2% to 9.9%, and thyroid disorders range from hypothyroidism to thyroid malignancies including autoimmune thyroiditis [3].

Thyroid hormone and its role in the development of the nervous system is well known. As per the previous reviewer, thyroid deficiency at an early age leads to various CNS manifestations like lethargy, poor feeding, delayed developmental milestones, and mental retardation [4]. A few animal studies have shown that brain-derived neurotrophic factor (BDNF) might be involved, as its expression in the limbic system is significantly reduced in offspring of rats treated with propylthiouracil (PTU) during pregnancy. Even in adult hypothyroid patients, a few studies have reported a reduction in cognition and memory [5,6]. In addition to these, studies have also shown that BDNF expression in the amygdala and hippocampus is drastically reduced in these patients [7,8]. Very few human studies are available correlating levels of BDNF and cognition in patients with hypothyroidism [9,10]. So, this review aims to explore the relation between BDNF and cognition in hypothyroid patients.

## Review

BDNF is a neurotrophin that is essential for neuron maintenance and differentiation. It regulates the neural transmission across both excitatory correlating levels of BDNF and cognition in patients with hypothyroidism inhibitory synapses [11]. Transcription, translation, and post-translational modifications all influence BDNF expression. The presence of a complex multi-level regulatory system emphasizes the importance and diversity of BDNF functions. Multiple promoters control transcription, resulting in activity-dependent and tissue-specific expression. At least four BDNF promoters have been identified in the rat, each driving the transcription of messenger ribonucleic acids (mRNAs) containing one of the eight non-coding exons spliced to the common 30 coding exons, resulting in a diverse population of BDNF transcripts. Epigenetic mechanisms can regulate the expression of specific BDNF exons, implying that environmental experiences proactively impact mature BDNF levels [12]. BDNF levels are high in the hippocampus, amygdala, cerebellum, and cerebral cortex. Hippocampal neurons have the highest concentration of all [13].

BDNF is synthesized as the precursor proBDNF, which can be stored in dendrites or axons before being cleaved intra- or extracellularly to produce a mature BDNF protein. BDNF is released as a concoction of pro and mature BDNF which are entirely activity-dependent. Interestingly, BDNF and proBDNF have contrasting effects on cellular function, adding to the complexities of BDNF protein function. Under both pathological and non-pathological conditions, proBDNF is secreted. proBDNF binds the neurotrophin receptor p75 (p75 NTR) receptor preferentially, facilitating long-term depression (LTD), and inducing apoptosis. BDNF, on the other hand, selectively binds to tyrosine kinase receptors (TrkB) and enhances cell viability, long-term potentiation (LTP), and spine complexity in its mature form. Thus proBDNF can be considered a regulatory component of BDNF activity in non-pathological conditions [14-16]. Both neurons and glia, including myelin-forming oligodendrocytes, have been shown to express BDNF and its receptors. TrkB truncated isoforms, which are expressed by astrocytes, have the ability to induce increases in intracellular calcium levels rather than established intracellular kinase pathways [17].

BDNF's importance in neuronal function has long been recognized. The research into its role in glial cell functions like myelination started only a few years ago and has been expedited since the mid-1990s when the BDNF knockout (KO) mouse was created [18]. BDNF KO mice had significantly lower expression levels of a critical myelin protein, myelin basic protein (MBP), as well as decreased levels of mRNA transcripts of MBP and proteolipid protein (PLP) in the hippocampus and cortex [19]. A study shows that even after cutting the proportion of myelinated axons in the optic nerve in two-week-old BDNF KO mice there was no change in the number of retinal ganglion cells or in the size or organization of the retinal layers. This proposes that BDNF promotes myelination rather than neuronal development [20].

**Figure 1:**
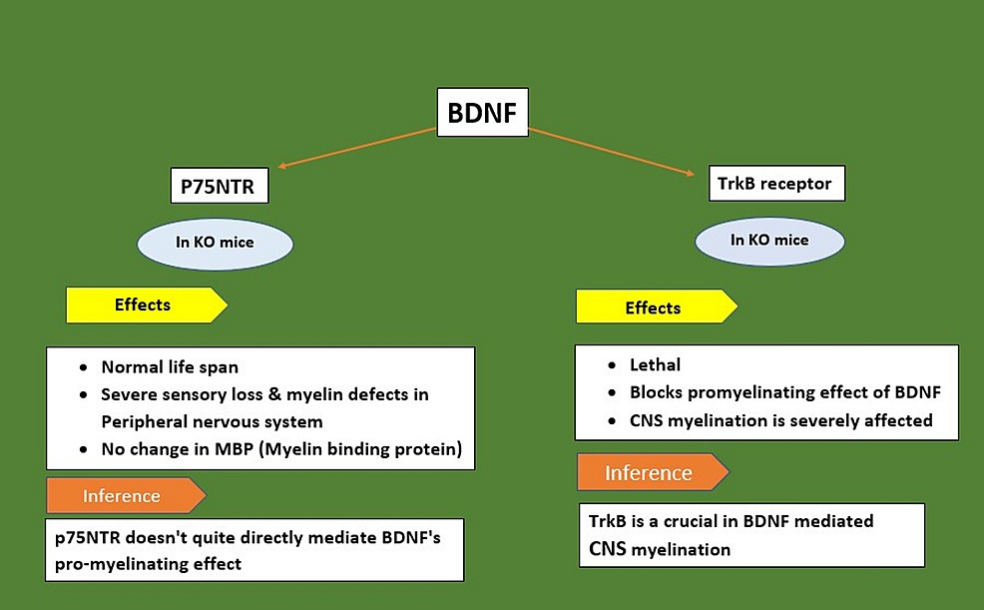
Receptors involved in brain-derived neurotrophic factor (BDNF) action on CNS myelination p75 NTR: neurotrophin receptor p75, TrkB: tyrosine kinase receptor, KO: knock out, CNS: central nervous system

BDNF heterozygous (HET) mice exhibit substantial reduction in the expression of myelin proteins such as MBP, PLP, myelin associated glycoprotein (MAG), and myelin oligodendrocyte glycoprotein (MOG) in the forebrain, corpus callosum, spinal cord, and optic nerves. This implies that endogenous BDNF boosts normal CNS myelination during development. According to in vitro studies, extracellular BDNF immensely boosts myelin formation in myelinating co-cultures of dorsal root ganglion neurons and OPCs compared to controls [21-22]. Receptors involved in BDNF action on CNS myelination are depicted in Figure 1 [23-24].

Despite limited evidence that BDNF has a strong influence on oligodendroglial proliferation, it is universally believed that it promotes myelin synthesis in vitro and in vivo by signaling through oligodendrocyte-expressed TrkB [25-26]. The creation of conditional TrkB knockout mice, in which TrkB has been deactivated from oligodendrocytes under the grasp of the MBP promoter, was crucial in understanding the role of BDNF-TrkB signaling in the myelinating process. During development, the density and size of myelinated axons in these mice were perfectly normal. The resulting myelin, however, was significantly thinner [26]. These findings imply that BDNF-TrkB signaling in oligodendroglia has no effect on the oligodendrocyte's first contact with the axon. Alternatively, by adulthood, myelin protein expression normalizes, with a particular consequence on the rate of ensheathment. Surprisingly, the phenotype of MBP conditional TrkB KO mice differs from that of BDNF HET mice, indicating that TrkB signaling in another cell type(s) affects the early events of myelination [21].

Despite the fact that the innate programme of myelin development occurs totally independent of nervous system activity, there is a lot of evidence that activity-dependent, plastic changes in myelin-forming cells influence myelin structure and neurological function. Also, complementary and likely temporally overlapping activity-independent and activity-dependent forms of myelination are forming the foundation for a model of myelin plasticity with comprehensive implications for neurological function in health and disease [27].

Surprisingly, evidence from various researchers suggests that myelination is more of a dynamic process than the classic model of innate myelination alone. Although oligodendrocytes have the innate power to direct myelination and elements of the myelin sheath, recent evidence suggests that intrinsic signs alone are unable of producing physiological myelin profiles seen in the CNS [28]. Adaptive myelination contributes to the formation of an innate myelin architecture that is patterned throughout life. The adaptive model shows how an animal's experience and neuronal activity impacts the cellular and ultrastructural properties of myelin [29].

BDNF expression is controlled by neural activity, that includes both sensory and environmental experiences. Both the transcription of the BDNF gene and the transport of BDNF mRNA and protein into the dendrites are regulated by neuronal activity, which might also encompass the activation of presynaptic N-methyl-D-aspartate (NMDA) glutamate receptors [30-31].

BDNF modulates neural plasticity in the adult brain by altering levels of NMDA receptors and regulating their phosphorylation, trafficking eventually leading to its upregulation [32], which enhances the synaptic strength. As BDNF has a crucial role in LTP it forms an essential part of memory formation and maintenance [33]. Also, BDNF increases the number, size, and complexity of dendrites through upregulated actin polymerization [34]. Various studies have documented the role of BDNF in learning and memory, plastic changes that are seen in spatial and recognition memory are mediated by BDNF [9,10].

Neurological manifestations in hypothyroidism

Hypothyroidism is the most common disorder among all thyroid disorders. Congenital hypothyroidism is the most exigent condition as it requires to be diagnosed at early stages and intervened, or else it leads to various neurological and developmental disorders [35]. Various research has shown that congenital hypothyroidism is more prevalent in India, with one out of every 2640 newborns affected with congenital hypothyroidism. The overall prevalence of hypothyroidism is 3.9% in India, of which subclinical is more predominant affecting females more than males [36].

Thyroid function has been shown to be important not only in cognitive development, but also in other aspects of nervous system activity, either directly influencing mechanisms involved in intrinsic regulatory circuits or indirect means through systemic effects. Because of the close relationship between the thyro-metabolic state and nervous system function, thyro-metabolic state disturbances are associated with a wide range of neurological signs and symptoms, including mood and cognitive disorders, headache, ophthalmoplegia, tremor and other movement disorders, muscle weakness, and so on [37].

It is evident that the thyroid hormone has a significant role in the development of the nervous system. Severe hypothyroidism in the neonatal period leads to developmental and cognitive impairments due to reduced mRNA and protein expression of BDNF in the hippocampus, cerebral cortex and cerebellum [5,6]. A few hypothyroid adult patients have also reported neuropsychiatric manifestations like anxiety, depression and reduced cognition [38-39]. Out of all the cognitive components, memory is the one that is majorly impaired as evident by imaging studies of the brain which showed reduced hippocampal volume and cerebral blood flow in regions which are involved in attention, visuospatial processing, working memory, and motor speed [40-42].

Various research, especially animal studies, suggests that thyroid hormones are essential for brain development. The exact underlying mechanism is not clear yet, but evolving evidence suggests that lack of BDNF expression might be one of the reasons, as this neurotrophin is vital for the formation of synapses, memory, etc. [6,43-44].

Hypothyroidism in adults if untreated leads to severe cognitive impairment [9]. It is observed that the hippocampus, parahippocampus, and amygdala show anatomical changes in hypothyroidism like decreased size of the hippocampus, decreased number of neurons in CA1 and CA3 regions, and loss of dendrites, which leads to reduced memory and learning behavior [9,10]. These changes are studied well in animals, but human studies on this aspect are sparse [45].

The hippocampus has a rich expression of thyroid hormone receptors, which might be the reason why hypothyroidism leads to impaired functions of the hippocampus, especially related to learning and memory [46]. According to Gilbert et al., different brain regions require thyroid hormones at different stages of development [47]. Studies have also suggested that thyroid hormones are involved in neurogenesis in the hippocampus [48].

There have been very few human studies that have documented reduced hippocampal volume as measured by magnetic resonance imaging (MRI) [49]. There was also a decrease in white and grey matter volumes measured by voxel-based morphometry. A decrease in grey matter was found in the bilateral cerebellum and left post-central gyrus, while a decrease in white matter was found in the bilateral cerebellum, right precentral gyrus, right inferior and middle frontal gyrus, right inferior occipital gyrus, and right inferior temporal gyrus [50]. Not many studies have correlated the reduced volumes to the extent of cognitive impairment in adult-onset hypothyroidism, however, Wheeler et al. demonstrated that children and adolescents with hypothyroidism have smaller hippocampi, reflected in reduced performance in various memory tasks [51].

Research evidence, especially from animal studies, has suggested that some of these changes in brain volumes are reversible once the appropriate therapy, i.e. hormone replacement therapy (HRT), is initiated [52]. Contradictory to this, some studies have also shown that these changes, especially the reduced number of neurons in CA1 and CA3 neurons in the hippocampus, did not improve even after the euthyroid state was achieved after HRT [53].

BDNF expression in hypothyroid patients

The role of BDNF in brain development, formation and maintenance of memory is well documented [2]. But does decreased thyroid hormone lead to reduced expression of BDNF? Various animal studies have documented reduced expression of BDNF in severe hypothyroidism, especially in offspring of rats that were treated with propylthiouracil (PTU) during pregnancy [5-8].

Increasing evidence suggests that BDNF is required for normal CNS development and also regulates synaptic transmission, dendritic structure, and synaptic plasticity in adults [54]. Human studies depicting the effect of hypothyroidism on BDNF expression are very rare. Studies have shown improvement in symptoms like depression and lethargy which are commonly found in hypothyroid patients after treatment with antidepressants, especially selective serotonin reuptake inhibitors (SSRIs) and after hormone replacement therapy [55]. This study has shown that thyroid hormone regulates 5HT neurotransmission by enhancing 5HT metabolism and 5HT receptor expression [56-57]. 5HT modulates the basal level of BDNF in the hippocampus and also contributes to stress-induced BDNF mRNA down-regulation in the hippocampus [58]. So, thyroid hormone modulates both 5HT and BDNF expression in the brain.

## Conclusions

Thyroid hormone has a substantial role in the development of the central nervous system, especially in aspects of cognition, learning and memory. These are evidently affected in neonatal hypothyroidism, and only a few studies have shown cognitive impairment even in adult hypothyroidism. The role of BDNF in formation and maintenance of memory is well established, and also thyroid hormones are necessary for BDNF expression in the hippocampus, parahippocampus and cerebral cortex which are well documented in animal studies and a few human studies. More human studies to correlate the levels and expression of BDNF in hypothyroidism are suggested to address this research gap. 

## References

[1] Kishosha PA, Galukande M, Gakwaya AM (2011). Selenium deficiency a factor in endemic goiter persistence in sub-Saharan Africa. World J Surg.

[2] Vanderpas JB, Contempré B, Duale NL (1990). Iodine and selenium deficiency associated with cretinism in northern Zaire. Am J Clin Nutr.

[3] Ogbera AO, Kuku SF (2011). Epidemiology of thyroid diseases in Africa. Indian J Endocrinol Metab.

[4] Chaker L, Bianco AC, Jonklaas J, Peeters RP (2017). Hypothyroidism. Lancet.

[5] Lasley SM, Gilbert ME (2011). Developmental thyroid hormone insufficiency reduces expression of brain-derived neurotrophic factor (BDNF) in adults but not in neonates. Neurotoxicol Teratol.

[6] Chakraborty G, Magagna-Poveda A, Parratt C, Umans JG, MacLusky NJ, Scharfman HE (2012). Reduced hippocampal brain-derived neurotrophic factor (BDNF) in neonatal rats after prenatal exposure to propylthiouracil (PTU). Endocrinology.

[7] Shafiee SM, Vafaei AA, Rashidy-Pour A (2016). Effects of maternal hypothyroidism during pregnancy on learning, memory and hippocampal BDNF in rat pups: beneficial effects of exercise. Neuroscience.

[8] Hashimoto K, Curty FH, Borges PP (2001). An unliganded thyroid hormone receptor causes severe neurological dysfunction. Proc Natl Acad Sci U S A.

[9] Heldt SA, Stanek L, Chhatwal JP, Ressler KJ (2007). Hippocampus-specific deletion of BDNF in adult mice impairs spatial memory and extinction of aversive memories. Mol Psychiatry.

[10] Cirulli F, Berry A, Chiarotti F, Alleva E (2004). Intrahippocampal administration of BDNF in adult rats affects short-term behavioral plasticity in the Morris water maze and performance in the elevated plus-maze. Hippocampus.

[11] Huang EJ, Reichardt LF (2001). Neurotrophins: roles in neuronal development and function. Annu Rev Neurosci.

[12] Hayes VY, Towner MD, Isackson PJ (1997). Organization, sequence and functional analysis of a mouse BDNF promoter. Brain Res Mol Brain Res.

[13] Timmusk T, Palm K, Metsis M (1993). Multiple promoters direct tissue-specific expression of the rat BDNF gene. Neuron.

[14] Lessmann V, Gottmann K, Malcangio M (2003). Neurotrophin secretion: current facts and future prospects. Prog Neurobiol.

[15] Pang PT, Teng HK, Zaitsev E (2004). Cleavage of proBDNF by tPA/plasmin is essential for long-term hippocampal plasticity. Science.

[16] Baker KB, Kim JJ (2002). Effects of stress and hippocampal NMDA receptor antagonism on recognition memory in rats. Learn Mem.

[17] Rose CR, Blum R, Pichler B, Lepier A, Kafitz KW, Konnerth A (2003). Truncated TrkB-T1 mediates neurotrophin-evoked calcium signalling in glia cells. Nature.

[18] Korte M, Carroll P, Wolf E, Brem G, Thoenen H, Bonhoeffer T (1995). Hippocampal long-term potentiation is impaired in mice lacking brain-derived neurotrophic factor. Proc Natl Acad Sci U S A.

[19] Djalali S, Höltje M, Grosse G (2005). Effects of brain-derived neurotrophic factor (BDNF) on glial cells and serotonergic neurones during development. J Neurochem.

[20] Cellerino A, Carroll P, Thoenen H, Barde YA (1997). Reduced size of retinal ganglion cell axons and hypomyelination in mice lacking brain-derived neurotrophic factor. Mol Cell Neurosci.

[21] Xiao J, Wong AW, Willingham MM, van den Buuse M, Kilpatrick TJ, Murray SS (2010). Brain-derived neurotrophic factor promotes central nervous system myelination via a direct effect upon oligodendrocytes. Neurosignals.

[22] Peckham H, Giuffrida L, Wood R (2016). Fyn is an intermediate kinase that BDNF utilizes to promote oligodendrocyte myelination. Glia.

[23] Cosgaya JM, Chan JR, Shooter EM (2002). The neurotrophin receptor p75NTR as a positive modulator of myelination. Science.

[24] Klein R, Smeyne RJ, Wurst W (1993). Targeted disruption of the trkB neurotrophin receptor gene results in nervous system lesions and neonatal death. Cell.

[25] Du Y, Fischer TZ, Lee LN, Lercher LD, Dreyfus CF (2003). Regionally specific effects of BDNF on oligodendrocytes. Dev Neurosci.

[26] Wong AW, Xiao J, Kemper D, Kilpatrick TJ, Murray SS (2013). Oligodendroglial expression of TrkB independently regulates myelination and progenitor cell proliferation. J Neurosci.

[27] Mount CW, Monje M (2017). Wrapped to adapt: experience-dependent myelination. Neuron.

[28] Marques S, Zeisel A, Codeluppi S (2016). Oligodendrocyte heterogeneity in the mouse juvenile and adult central nervous system. Science.

[29] Bechler ME, Byrne L, Ffrench-Constant C (2015). CNS myelin sheath lengths are an intrinsic property of oligodendrocytes. Curr Biol.

[30] Cowansage KK, LeDoux JE, Monfils MH (2010). Brain-derived neurotrophic factor: a dynamic gatekeeper of neural plasticity. Curr Mol Pharmacol.

[31] Branchi I, Karpova NN, D'Andrea I, Castrén E, Alleva E (2011). Epigenetic modifications induced by early enrichment are associated with changes in timing of induction of BDNF expression. Neurosci Lett.

[32] Caldeira MV, Melo CV, Pereira DB, Carvalho RF, Carvalho AL, Duarte CB (2007). BDNF regulates the expression and traffic of NMDA receptors in cultured hippocampal neurons. Mol Cell Neurosci.

[33] Bramham CR, Messaoudi E (2005). BDNF function in adult synaptic plasticity: the synaptic consolidation hypothesis. Prog Neurobiol.

[34] Rex CS, Lin CY, Kramár EA, Chen LY, Gall CM, Lynch G (2007). Brain-derived neurotrophic factor promotes long-term potentiation-related cytoskeletal changes in adult hippocampus. J Neurosci.

[35] Desai MP (1997). Disorders of thyroid gland in India. Indian J Pediatr.

[36] Usha Menon V, Sundaram KR, Unnikrishnan AG, Jayakumar RV, Nair V, Kumar H (2009). High prevalence of undetected thyroid disorders in an iodine sufficient adult south Indian population. J Indian Med Assoc.

[37] Stasiolek M (2015). Neurological symptoms and signs in thyroid disease. Thyroid Res.

[38] Samuels MH (2014). Psychiatric and cognitive manifestations of hypothyroidism. Curr Opin Endocrinol Diabetes Obes.

[39] Easson WM (1966). Myxedema with psychosis. Arch Gen Psychiatry.

[40] Correia N, Mullally S, Cooke G (2009). Evidence for a specific defect in hippocampal memory in overt and subclinical hypothyroidism. J Clin Endocrinol Metab.

[41] Miller KJ, Parsons TD, Whybrow PC (2007). Verbal memory retrieval deficits associated with untreated hypothyroidism. J Neuropsychiatry Clin Neurosci.

[42] Bauer M, Silverman DH, Schlagenhauf F (2009). Brain glucose metabolism in hypothyroidism: a positron emission tomography study before and after thyroid hormone replacement therapy. J Clin Endocrinol Metab.

[43] Thorpe-Beeston JG, Nicolaides KH, McGregor AM (1992). Maternal and Fetal Thyroid Function in Pregnancy. Thyroid.

[44] Kasatkina EP, Samsonova LN, Ivakhnenko VN, Ibragimova GV, Ryabykh AV, Naumenko LL, Evdokimova YA (2006). Gestational hypothyroxinemia and cognitive function in offspring. Neurosci Behav Physiol.

[45] Cooke GE, Mullally S, Correia N, O'Mara SM, Gibney J (2014). Hippocampal volume is decreased in adults with hypothyroidism. Thyroid.

[46] de Jong FJ, den Heijer T, Visser TJ, de Rijke YB, Drexhage HA, Hofman A, Breteler MM (2006). Thyroid hormones, dementia, and atrophy of the medial temporal lobe. J Clin Endocrinol Metab.

[47] Gilbert ME, Rovet J, Chen Z, Koibuchi N (2012). Develop- mental thyroid hormone disruption: prevalence, environ- mental contaminants and neurodevelopmental consequences. Neurotoxicology.

[48] Desouza LA, Ladiwala U, Daniel SM, Agashe S, Vaidya RA, Vaidya VA (2005). Thyroid hormone regulates hippocampal neurogenesis in the adult rat brain. Mol Cell Neurosci.

[49] Oatridge A, Barnard ML, Puri BK, Taylor-Robinson SD, Hajnal JV, Saeed N, Bydder GM (2002). Changes in brain size with treatment in patients with hyper- or hypothyroidism. AJNR Am J Neuroradiol.

[50] Singh S, Modi S, Bagga D, Kaur P, Shankar LR, Khushu S (2013). Voxel-based morphometric analysis in hypothyroidism using diffeomorphic anatomic registration via an exponentiated lie algebra algorithm approach. J Neuroendocrinol.

[51] Wheeler SM, Willoughby KA, McAndrews MP, Rovet JF (2011). Hippocampal size and memory functioning in children and adolescents with congenital hypothyroidism. J Clin Endocrinol Metab.

[52] Ambrogini P, Cuppini R, Ferri P (2005). Thyroid hormones affect neurogenesis in the dentate gyrus of adult rat. Neuroendocrinology.

[53] Koromilas C, Liapi C, Schulpis KH, Kalafatakis K, Zarros A, Tsakiris S (2010). Structural and functional alterations in the hippocampus due to hypothyroidism. Metab Brain Dis.

[54] Binder DK, Scharfman HE (2004). Brain-derived neurotrophic factor. Growth Factors.

[55] Vaidya VA, Castro ME (2001). Influence of thyroid hormone on 5-HT(1A) and 5-HT(2A) receptor-mediated regulation of hippocampal BDNF mRNA expression. Neuropharmacology.

[56] Mason Mason, Bondy Bondy, Nemeroff Nemeroff (1987). The effects of thyroid state on beta-adrenergic and serotonergic receptors in rat brain. Psychoneuroendocrinology.

[57] Sandrini M, Vitale G, Vergoni AV (1996). Effect of acute and chronic treatment with triiodothyronine on serotonin levels and serotonergic receptor subtypes in the rat brain. Life Sci.

[58] Vaidya VA, Terwilliger RZ, Duman RS (1999). Role of 5-HT2A receptors in the stress-induced down-regulation of brain-derived neurotrophic factor expression in rat hippocampus. Neurosci Lett.

